# The late arrival of coronavirus disease 2019 (COVID-19) in Chad: mitigating spread in Ndjamena focused on specific target population

**DOI:** 10.11604/pamj.2020.37.338.26143

**Published:** 2020-12-13

**Authors:** Zita Aleyo Nodjikouambaye, Chatté Adawaye, Fissou Henry Yandai, Laurent Bélec

**Affiliations:** 1 Regional Doctoral School of Tropical Infectious Disease, Franceville, Gabon,; 2 Mobile Laboratory for Hemorrhagic and Respiratory Viruses in Ndjamena, Ndjamena, Chad,; 3 National Higher Institute of Sciences and Techniques in Abéché, Abéché, Chad,; 4 Laboratory of Virology, European Hospital Georges Pompidou and University of Paris, Paris, France

**Keywords:** SARS-CoV-2, COVID-19, epidemiology, Chad

## Abstract

The novel coronavirus disease 2019 (COVID-19) has rapidly spread to all 7 continents. Due to yet unknown reasons, the African continent has remained relatively unaffected, especially Chad. We discuss the importance of mitigating spread in Ndjamena focused on specific target population.

## Opinion

The COVID-19 pandemic is an emerging infectious disease, called coronavirus disease or COVID-19, caused by the SARS-CoV-2 coronavirus, which appeared in Wuhan on November 17, 2019, in the province of Hubei (in central China), before spreading around the world. The World Health Organization (WHO) first alerts the People's Republic of China and its other member states, and then declared a Global Public Health Emergency of International Concern on January 30, 2020. Since then, COVID-19 has spread to all continents. As of 12 September 2020, 28,815,191 cases of infection have been officially diagnosed since the start of the epidemic, with 925,989 deaths in the worldwide. Less impacted than the rest of the world, the African continent has so far recorded 1,338,020 cases with 32,341 deaths according to the African Union Center for Disease Prevention and Control. But the signs indicating that this greatly underestimated balance sheet is increasing. In anticipation of importation of cases to Africa, the WHO, the Africa Centers for Disease Control and Prevention (CDC), all national governments, and several public health organizations have spent 2 months scaling up preparedness efforts to control the spread of the COVID-19 pandemic throughout Africa [1-3]. This support is critical to allow African governments to implement the International Health Regulations Emergency Committee recommendations.

**Figure 1:**
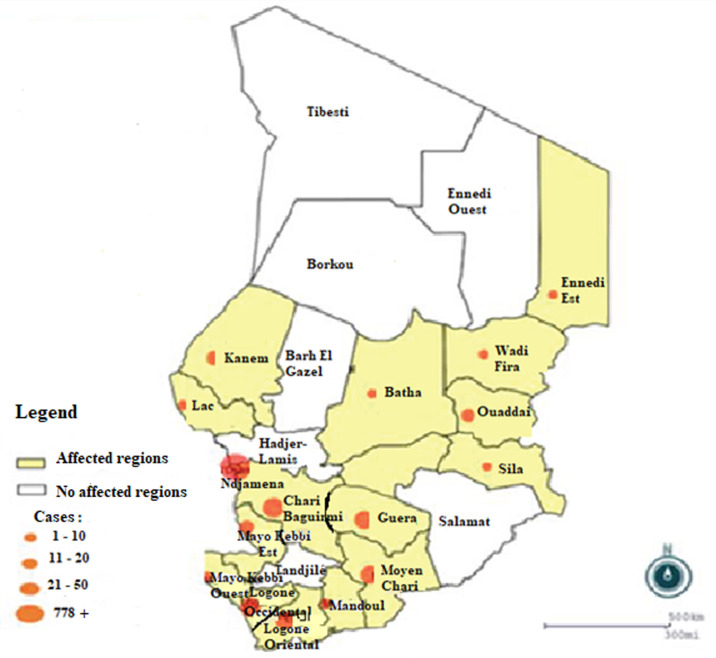
evolution of confirmed cases of COVID-19 in Chad

**Figure 2:**
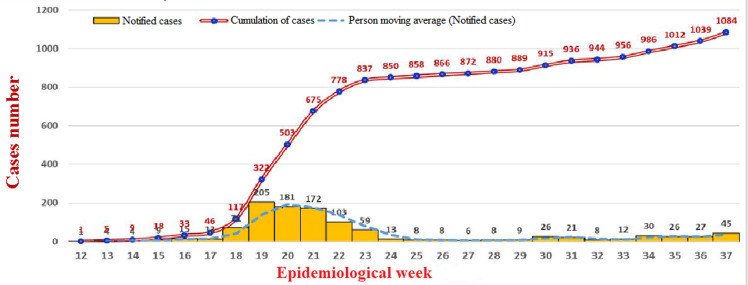
geographical distribution of cases in Chad

The COVID-19 epidemic in Chad was declared on 19 March 2020 by the Government following confirmation by the laboratory of a first case, which arrived on 15 March 2020 by the TCHADIA Airlines flight from Cameroon. The following 9 cases were imported cases. A first local transmission was reported on 6 April 2020 in Ndjamena which a domestic transmission. To date, on 12 September 2020, Chad has 1,084 confirmed cases with 80 deaths in 16 provinces [4] (Figure 1). Most of cases are in Ndjamena, the capital of Chad. A total, 1018 confirmed cases recovered and 66 under treatment; 21,984 people were maintained in quarantine, including 20,523 students from affected countries across 16 provinces. Chad does not have adequate capacity for polymerase chain reaction testing to confirm SARS-CoV-2 in suspected cases. The mobile laboratory for hemorrhagic and respiratory viruses in Ndjamena, Chad quickly has adapted its equipment to be able to test samples for COVID-19 with WHO and its partners support. To avoid excessive morbidity and mortality from uncontrolled COVID-19, greater resources and new approaches to strengthen health systems have been put in place by authorities. After the confirmation of the first case of COVID-19, the authorities implemented measures banning gatherings of more than 50 people. Schools, universities and non-food businesses were closed. However, the screening strategy has changed considerably since the start of the epidemic. To date, there are 14 laboratories distributed in different regions for screening. This strategy made it possible to detect all suspected cases, hence the increase in confirmed cases during the last two months (Figure 2). In addition, the spread of COVID-19 in Chad clearly represents an additional concern as the country already suffers from several public health problems (HIV with a prevalence of 1.6%, hepatitis, malaria, acute respiratory infections and diarrheal diseases). Malnutrition affects 44.2% of the population (WHO, Cooperation Strategy 2017-2020). It should be noted that risk factors for more serious diseases and fatal outcomes include older age (> 60 years) and co-morbidities such as cardiovascular disease, chronic lung disease, diabetes or cancer, which are also common in aging patients with HIV and hepatitis. Therefore, it can be reasonably assumed that many people in Chad will be at high risk of COVID-19.

To date, there are numerous trials currently evaluating therapeutic interventions targeting the SARS-CoV-2 virus and its damaging immune response in the lungs, which causes acute respiratory distress syndrome and is a major cause of death [5]. The main treatment used for COVID-19 is remdesivir, an antiviral drug originally designed to treat Ebola virus disease and Marburg virus infection. There remains an urgent need to conduct randomized clinical trials of adequate power to confirm the effectiveness of treatments against COVID-19. In the absence of an effective treatment or vaccine, traditional epidemic control measures must be strengthened in Chad, including social distancing, frequent hand washing, coughing and sneezing, and avoiding touching each other, eyes, nose and mouth. Chad known for its reputation as a community, and many daily activities revolve around social interactions, including shopping at church or at the market. Social distancing has been particularly difficult, especially for people looking for food or needing a daily salary. Innovative strategies to reduce social interactions began immediately after the first case was confirmed. Prevention in community settings emphasizes individual and collective practices. Television, religious services (via radio, television, and social media), radio ads, toll-free COVID-19 hotlines and local leaders have been mobilized to educate the public. The Chadian authorities in collaboration with the WHO have put in place travel bans for nationals from countries with high COVID-19 endemics and complete lockdowns including screening at points of entry into the country, prevention and control of infections in health facilities, clinical management of people with severe COVID-19 infection.

In late June, the country began to ease the measures that had until then been applied to fight the spread of the virus. The reopening of the airport area or any passenger arriving in Chad must be provided with a negative polymerase chain reaction (PCR) test dating less than 7 days and is quarantined for 7 days with the requirement of a new negative test to exit the quarantine. The reopening of places of worship as well as exam and university classes, closed for three months. However, Chad must continue to provide and mobilize additional funds for essential health services during the COVID-19 pandemic, particularly tuberculosis, malaria, HIV and maternal and child health. The delivery of prevention, diagnosis, treatment and person-centered care must be provided in parallel with the response to COVID-19. Since the modes of transmission of tuberculosis, COVID-19 and other respiratory tract infections are similar, measures must be put in place to limit the transmission of tuberculosis and COVID-19 in collective structures and health establishments. The provision of personal protective equipment to life-threatening healthcare workers needs to be prioritized and strengthened. Accurate diagnostic tests are essential for all respiratory tract infections (including COVID-19) and require a continuous supply of diagnostic tests. Existing Pan-African networks for emerging and re-emerging diseases that work in close relationship with the Africa CDC and the WHO regional office will play a crucial role in mitigating the impact of COVID-19. Existing tuberculosis laboratory networks and mechanisms for the transport and processing of specimens should be used for the diagnosis and surveillance of COVID-19.

In conclusion, the global burden continues to increase in Chad. The effects of temperature and humidity play an important role in the spread of respiratory viral infections during what is commonly referred to as the “flu” season caused by the influenza virus [6]. It is still unclear whether this will be effective for SARS-CoV-2 or to what extent the seasons contribute to the transition from an epidemic to a pandemic. However, if COVID-19 is not contained, it is possible that countries in the southern hemisphere such as South Africa, Brazil and Australia will experience increased intensities of infection during their months of winter between May and September 2020 [7,8]. Data from China has shown that more than 99.0% of people aged from 1 to 70 years who contract COVID-19 recover without specific treatment and generate herd immunity. Most will have cold/flu symptoms and will not even know they are infected. An effective public health message on COVID-19 should align with the political and scientific briefings of the Chadian government, and this should focus on education as well as securing the public. Elderly patients with co-morbidities or those with immunosuppression are most at risk, and we must work to prevent infection in these populations.

## References

[1] Kapata N, Ihekweazu C, Ntoumi F, Raji T, Chanda-Kapata P, Mwaba P (2020). Is Africa prepared for tackling the COVID-19 (SARS-CoV-2) epidemic. Lessons from past outbreaks ongoing pan-African public health efforts, and implications for the future. Int J Infect Dis.

[2] Largent EA (2016). EBOLA and FDA: reviewing the response to the 2014 outbreak, tofind lessons for the future. J Law Biosci.

[3] World Health Organization (2020). Weekly epidemiological update. Data as received by WHO from national authorities as of 10 am CEST.

[4] Organisation mondiale de la santé Rapport de la Situation Épidémiologique COVID-19 au Tchad No. 173.

[5] U.S National Library of Medicine COVID-19 Studies Listed on ClinicalTrials.gov (Beta).

[6] Luo W, Majumder MS, Liu D, Poirier C, KD Mandl, Lipsitch M (2020). The role of absolute humidity on transmission rates of the COVID-19 outbreak.

[7] Infection Control Africa Network (ICAN) Online training ECHO programmed.

[8] Center for Communicable Disease Dynamics Seasonality of SARS-CoV-2: Will COVID-19 go away on its own in warmer weather?.

